# Development of Structural Model on Suicidal Ideation in Adolescents’ Exposure to Violence

**DOI:** 10.3390/ijerph18063215

**Published:** 2021-03-19

**Authors:** Jeoung Mi Kim

**Affiliations:** Department of Nursing Science, KAYA University, Gimhae 50830, Korea; jeoung66@kaya.ac.kr

**Keywords:** suicidal ideation, adolescent, violence

## Abstract

Purpose: This study developed and examined a structural model and influencing factors of suicidal ideation by path analysis of family violence exposure, school violence exposure, anger, aggression, depression, hopelessness, and ego resilience among adolescents. Methods: A hypothetical model was constructed on the basis of general strain theory developed by Agnew, as well as a review of studies in the literature related to suicidal ideation in adolescents in terms of violence exposure. The subjects were 1150 middle school students located in P city and K province. The model included 8 concepts and 24 paths. Data were collected using self-report questionnaires from September 2 to 20, 2013, and analyzed using the IBM SPSS and AMOS 21.0 programs. Results: Family violence exposure, school violence exposure, anger, depression, hopelessness, and ego resilience showed a direct effect, while aggression showed an indirect effect on suicidal ideation in adolescents. These factors accounted for 45% of the variance of suicidal ideation in middle school students in terms of violence exposure. Conclusions: The results suggest that suicidal ideation of adolescents who are exposed to violence could be decreased by increasing ego resilience and reducing family violence exposure. It is necessary to develop an intervention strategy to prevent suicidal ideation.

## 1. Introduction

Suicidal ideation is considered the explicit desire to kill oneself. “Suicidal ideation” refers to ideation that life is not worth living, ranging in intensity from fleeting ideation through to concrete, well thought-out plans for killing oneself, or self-destruction [1]. The suicide rate of adolescents has been increasing every year and it is one of the most important social issues in South Korea. The suicide rate of adolescents decreased from 7.7 to 6.5 people per 100,000 people over 10 years as per the Organization for Economic Co-operation and Development (OECD) nations [2]. On the other hand, South Korea’s adolescent suicide rate was 6.4 in 2001 and increased to 9.4 in 2011, showing a total of 47% increase [3]. A survey reported that 42.8% of middle and 35.5% of high school students had suicidal ideation among 1750 school students in Seoul [4]. Even a minimum level of suicidal ideation develops into a serious level or leads to attempt suicide. Therefore, suicidal ideation and suicide attempts of adolescents are a serious problem that cannot be overcome and require special attention.

Socio-environmental conditions are also considered causes of adolescents’ suicidal ideations, such as family and the school [5,6]. Family and school violence have been reported as associated factors with adolescents’ suicidal tendency [7]. Middle school students need to be more careful than any other age group because the risks of suicidal ideation are higher amongst them than high school students [8]. The National School Violence Survey found that 12.0% of youths suffered from school violence for a year, and 44.7% of them committed suicide due to school violence. In addition, 5.7% of high school students experienced school violence compared to 13.4% of middle school students [9].

The general strain theory explains that adolescents fail to socialize when they fail to internalize social norms, leading to extreme deviant behavior and suicide when strains persist in everyday life. Strain factors lead to negative emotional reactions such as anxiety and depression, and negative emotional reactions have been identified as a major cause of suicide [10]. Children who have witnessed or experienced violence at home may experience internalization problems of emotional anxiety and psychological and cognitive maladaptation, and externalization problems such as aggression and misconduct [11]. In addition, violence in schools, such as bullying and assault, add to the strain, stress, depression, and hopelessness of adolescents [12].

Most previous studies have shown that the relationship between child abuse, school violence, and suicide, as well as partial exposure, such as violence, anger, aggression, and depression [13]. The child abuse, violence, and experiences of physical abuse by parents at home are reported to be highly related to anger, aggression, and suicide [4,5,6,7,9]. It induces highly negative thoughts and influences social support among middle and high school students; therefore, the risk of suicidal thoughts may increase [6,8]. In addition, a national survey on school violence found that 12.0% of teenagers reported the experience of school violence for about 1 year, and 44.7% of them had suicidal thoughts [9].

However, there is a lack of studies explaining the effects of anxiety, aggression, depression, and suicidal ideation and direct and indirect effects, such as anxiety, aggression, depression, and suicidal ideation [14]. According to the general strain theory, strains may be adaptive or non-adaptive, and everyone does not feel strain in same pattern but it depends on the characteristics of the condition such as environmental variables surrounding the individual [15]. This study sought to find a mechanism that can cope with crisis or strain, with resilience referring to overcoming negative life events through a multidimensional process consisting of individual factors and relationship factors. 

Research has suggested self-resilience as an internal variable to cope with stress situations and shows that adaptability such as self-destructive behavior is in accordance with the individual’s ego resilience, reporting that ego resilience has an interaction and moderating effect between daily life (school life, self-related problems, and environmental problems) and suicidal ideation [16]. On the basis of the review, the researchers found that ego resilience can be predicted in that that there are some important factors influencing social responses. 

Agnew’s general strain theory (GTS) has drawn attention as a model for integrated flight causes, described in previous studies as explaining the relationship between strain and suicide risk [17]. This study planned a theoretical framework for developing nursing intervention programs to assess the adolescent suicidal risks, as well as to predict and mediate risk groups.

### Conceptual Model and Hypothetical Model

**Figure 1:**
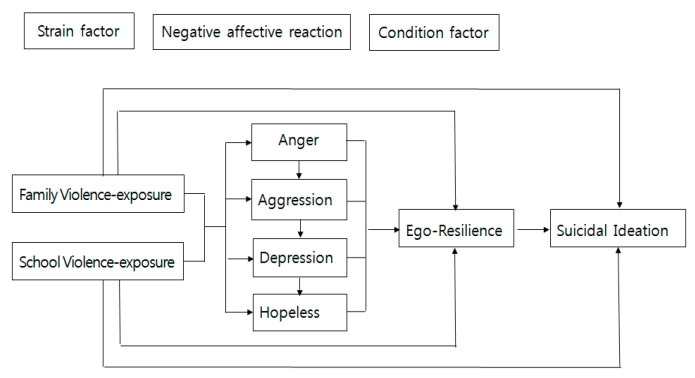
Conceptual framework.

Agnew’s [15] general strain theory (GTS) has mainly been used to explain delinquency or deviation, but the key factor of general tension theory, the tension factor, ahs been linked to negative emotional reactions such as anger, frustration, depression, and aggression. On the basis of previous studies [17,18] explaining the relationship that increases the risk of suicide, researchers have established exposure to domestic violence and school violence as tension factors that can induce suicidal thoughts. Depression and hopelessness were included as negative emotional reactions caused by tension, and self-elasticity was set as a conditional factor in terms of having a significant effect on suicidal thoughts (Figure 1).

**Figure 2:**
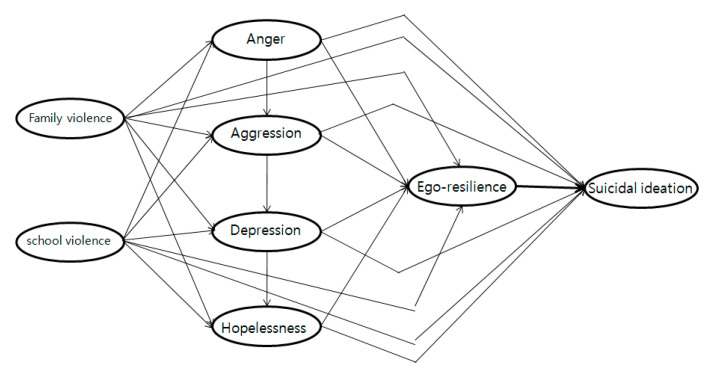
Hypothetical model.

The hypothetical model of this study consists of two exogenous and six endogenous variables. Exogenous variables were exposure of school violence and family violence. Endogenous variables were anger, aggression, depression, hopelessness, ego resilience, and suicidal ideation. Exogenous variables were affecting the mediating endogenous variables. In addition, the final endogenous variable affects suicidal ideation and ego resilience affects the final endogenous suicide ideation (Figure 2).

The purpose of this study was to develop a hypothesis model based on previous studies and predict the influencing factors of suicidal ideation among adolescents, ultimately finding a cause-and-effect path among the selected variables. In addition to analysis, the paths by which violent exposure explains the suicidal ideation of adolescents. 

## 2. Materials and Methods

### 2.1. Study Design

The present study utilized a cross-sectional research design to construct and verify the structural model by general strain theory [18] and inferences from previous studies to determine the suicidal ideation of adolescents through violence exposure.

### 2.2. Settings and Sample

The study sample comprised middle school students from P city and K city, Korea. Participants were selected by convenience sampling; the survey was administered in a classroom setting during a regularly scheduled class period with standardized instructions. The sample size was 5–10 samples per 1 estimation parameter and the estimated parameters were 195 × 5 = 975 or more. This study selected 1220 adolescents to ensure a stable sample.

### 2.3. Research Instrument

#### 2.3.1. Exposure to Family violence

Exposure to family violence tool witnessing child abuse and marital violence, the CTS2, was made by modifying and supplementing the existing CTS (conflict tactics scale) by Straus [19]. The CST2 tool was revised and supplemented by Baik [20] with 32 items for children violence. In this study used 19 questions of psychological violence (8 questions) and physical violence (11 questions). Scores were given as 0 (not exposed) and 1 (exposed) according to the presence or absence of exposure to violence, and the higher the score, the higher the exposure to domestic violence. Reliability of subscales of CTS2 Cronbach’s α score for psychological violence = 0.83 and physical violence = 0.92 in this study.

The child abuse tool refers to verbal abuse and physical abuse. The verbal abuse tool was developed on the basis of 15 commonly used forms of verbal abuse by parents towards their children and was modified by Yeon [21]. Physical abuse tool was used in this study, which was modified by Kim and Lee [22] scores of the tool 0 (no exposure) and 1 (exposure), the higher score indicates higher family violence exposure. Reliability score of Cronbach’s α= 0.84 for physical abuse and Cronbach’s α = 0.89 for verbal abuse.

#### 2.3.2. Exposure to School Violence

The bullying scale was developed by Lee and Kwak [23] and modified by Jeong and Ahn [24], with 7 types of victims of school violence such as body violence, extortion, threats and intimidation, verbal abuse, harassment, bullying, and cyber bullying. In addition, 2 sub factors (school violence damage 1 and school violence damage 2) were used to increase the reliability and validity of the tool as per the expert’s opinion. Cronbach’s α = 0.78 was used in this study.

#### 2.3.3. State–Trait Anger Inventory-Korean Version. STAXI-K

The state–characteristic anger scale was developed by Spielberger et al [25] and modified by Chon et al [26]. STAXI-K consists of a total of 20 items (10 state anger and 10 items of characteristic anger) and is measured by a 4-point Likert scale. In the study, Cronbach’s α = 0.82 for anger control; 0.89 for anger out; 0.86 for anger; and Cronbach’s α = 0.94, 0.89 and 0.91 for all types of anger.

#### 2.3.4. Aggression

The Peer Conflict Scale (PCS) produced by Marsee et al [27] was used by Han and Kim [28]. It consists of 4 categories comprising overt/relational aggression and reactive/proactive aggression, with 10 questions in each category. The tool is measured by 4-point Likert scale. The internal reliability of each domain of original tool was 0.76–0.90. In this study, Cronbach’s α = 0.76 for the leading external appearance, Cronbach’s α = 0.50 for overt relational aggression, 0.85 for reactive aggression, 0.80 for proactive aggression, and over all Cronbach’s α = 0.88 in this study.

#### 2.3.5. Depression

Center for Epidemiological Study- Depression CES-D is a depression scale produced by the National Institute of Mental Health (NIMH) to measure the level of depression in the general population. The Korean version of CES-D was used in this study, consisting of 20 questions, including depressive emotions positive emotions, isolation feelings in an interpersonal relationship. Each item was graded 0–3 (total physical deterioration of slowing behaviors at 60 points), with a minimum of 16 points. Cronbach’s α of depression scale was 0.90 in this study.

#### 2.3.6. Hopelessness

The Beck Hopeless Scale (BHS) developed by Beck et al [29] was translated into Korean by Shin et al. [30]. The BHS is a self-reporting questionnaire, consisting of 20 true or false items that measure positive and negative beliefs about the future. Some of the items are scored reverse-wise, and high score indicates higher level of hopelessness in total score from 0 to 20. Cronbach’s alpha for in this study was 0.88.

#### 2.3.7. Suicidal Ideation

The Scale of Suicidal Ideation (SSI) was developed by Beck et al. [29], and Park and Shin et al [30] modified and evaluated it through clinical interviews. Among the 19 items, 2 items were deleted due to low response rate. The sub-factors were extracted from the questionnaire package as suicide ideation 1 and suicide ideation 2. Cronbach’s α was 0.69 in this study

#### 2.3.8. Ego Resilience

The Ego Resiliency Scale (ER) was developed by Block and Kremen [31] and modified by Yoo and Shim [32]. This scale consists of 14 questions with 5 sub-factors. The tool is measured by a 5-point Likert scale and 1 to 14 points, with higher points indicating a higher ego resilience. In this study, Cronbach’s α was 0.77, vitality 0.61, emotional control 0.54, curiosity 0.84, and optimism 0.61.

### 2.4. Data Collection Process

The study was initiated after obtaining ethical approval. A letter was sent to home for parental consent; at the same time, the purpose of the study and data collection method were explained to the related grade and head of the institution before data collection, and subject recruiting was allowed. The researcher collected the parental and personal consent form from the students, also being explained them that they could withdraw at any time in case of inconvenience. The pilot study was conducted among 120 students at 4 middle schools in 2 cities, and content validity, reliability, and factor analysis were performed. Data were collected from September 2 to 20, 2013, during the break and leisure period with the help of class teachers and assistant workers. The survey was conducted for about 40 to 50 min and distributed a small complementary gift to the participants at the end of data collection. A total of 1220 pieces of data were collected, and 1150 were finalized due to incomplete questionnaires.

### 2.5. Data Analysis

The collected data were analyzed by IBM SPSS 21.0 and AMOS 21. software. The general characteristics of the subjects and major variables were analyzed by descriptive statistics. The correlations between variables were calculated using Pearson’s correlation coefficients. The factor extraction was performed by using Varimax Rotation method and the reliability was tested by Cronbach’s alpha coefficient. In the confirmatory factor analysis, a structural equation model analysis was performed to explore the structural relationships of the model. The goodness-of-fit model was analyzed using chi-square (x^2^) goodness of fit index (GFI) and adjusted goodness of fit index (AGFI), root mean square residual (RMR), root mean square error of approximation (RMSEA), comparative normed fit index (CNFI), and Tucker–Lewis index (TLI). The bootstrapping method was used to analyze the direct and indirect mediation effects of suicide ideation variables.

## 3. Results

### 3.1. General Characteristics of the Subject

The age of the subjects were between 13 and 14 years old, and most of them were male. The education of fathers and mothers was 41.9% and 53.1%, respectively. The monthly income of the family was 3–4 million won. In academic achievement, most of the students perceived as moderate. In terms of exposure to violence, the highest rate of students was exposed to family violence (65.3%) rather than school violence (22.0%). The descriptive statistics of the variables used in this study are shown in Table 1. 

### 3.2. Descriptive Statistics of Selected Varibles

In terms of the selected variables given in Table 1, this study found that skewness was closer to 0 and kurtosis score was 3; therefore, this confirmed the normality of the distribution of each variable. Some points of skewness score in domestic violence, school violence, anger and aggression exceeded the absolute value of 3; the absolute value of the kurtosis value exceeded 10, whereas the remaining variables did not. Accordingly, the data in this study were analyzed after solving the problem by bootstrapping.

The validity was evaluated through average variance extracted (AVE) and construct reliability (CR), which present the coincidence between latent variables and measurement variables in which the CR values of each construct concept were all 0.70 or more, which was significant. The AVE value of each construct concept value was 0.50 or more, confirming that this model had convergent validity (Table 1).

### 3.3. Correlation of Major Research Variables

The correlation of the variables showed that all variables used in this study had a significant correlation. The final outcome of the variables were that depression (*r* = 0.568, *p* < 0.01), hopelessness (*r* = 0.420, *p* < 0.01), anger (*r* = 0.389, *p* < 0.01), family violence exposure (*r* = 0.311, *p* < 0.01), school violence exposure (*r* = 0.248, *p* < 0.01), and aggression (*r* = 0.222, *p* < 0.01) showed positive correlations. Resilience was negatively correlated (*r* = −0.225, *p* < 0.01). Additionally, suicidal ideation was highly correlated with depression (*r* = 0.568, *p* < 0.01) and hopelessness (*r* = 0.547, *p* < 0.01). These variables were closely related to each other. Ego resilience was negatively correlated with family violence exposure, school violence exposure, anger, aggressiveness, depression, hopelessness, and suicidal ideation and highly correlated with hopelessness (*r* = −0.355, *p* < 0.01). The exposure of family and school violence were least correlated with anger, aggression, and hopelessness. The correlation coefficient can be multicollinearity when the absolute value is over 0.80 (Table 2).

### 3.4. Testing of Structural Model of Suicidal Ideation Adolescents

#### 3.4.1. Confirmatory Factor Analysis and Goodness-of-Fit Tests

**Figure 3:**
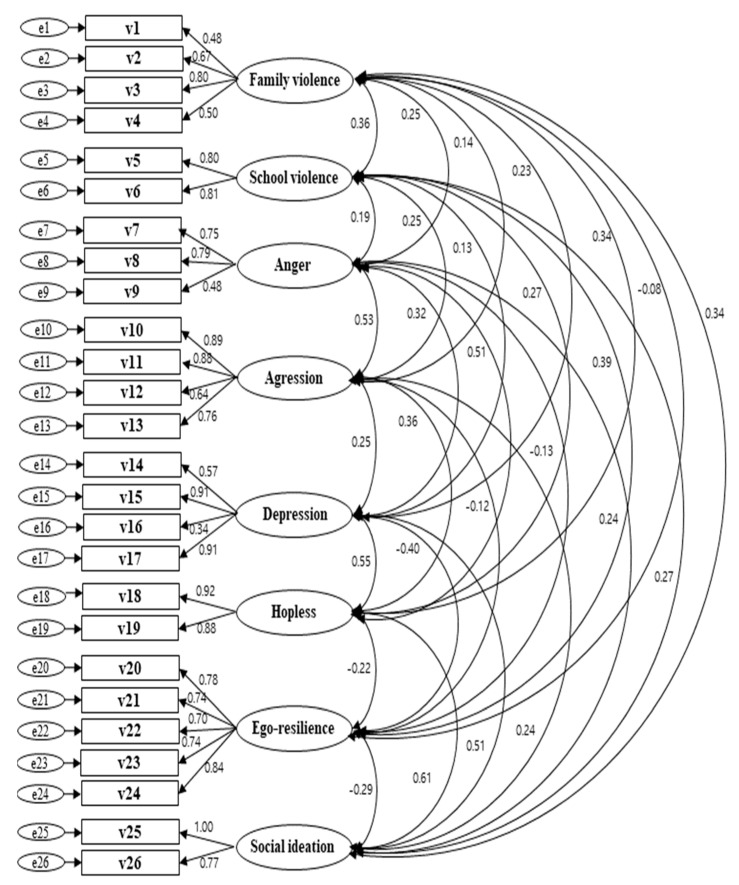
Confirmatory factor analysis.

Analyzed the data with structural equation modeling (SEM) techniques to test the hypothesized model (Figure 3). Prior to the analysis of the model, confirmatory factor analysis was performed to confirm the relationship between variables in the hypothetical model constructed on the basis of the theoretical background. As a result of confirmatory factor analysis, the hypothesized model revealed adequate fit to the data (χ^2^ = 1325.245 (df = 272, *p* < 0.01), CMIN/DF (/df) = 4.872, GFI = 0.921, AGFI = 0.898, NFI = 0.912, TLI = 0.915, RMSEA = 0.058, CFI = 0.929, RMR = 0.554) and was statistically significant (Table 3). In addition, all the path coefficients (factor variables) were statistically significant at the significance level of 0.01, indicating that the measurement variables reflected initial support for the model. To improve the fit of the model, we tested an alternative model to find out students’ variables.

#### 3.4.2. Analysis of Hypothetical Model

The goodness-of-fit index of the hypothetical model in this study was found to be χ^2^ = 1423.896 (df = 275, *p* < 0.01), CMIN/DF (/df) = 5.178, GFI = 0.915, AGFI = 0.891, NFI = 0.906, TLI = 0.908, RMSEA = 0.060, CFI = 0.922, RMR = 0.717. Specifically, the result of the goodness-of-fit analysis was 1423.896 (*p* < 0.01) and the hypothetical model was rejected. The goodness-of-fit could be evaluated between 2.0 and 3.0, results showed CMIN/DF in the goodness-of-fit was less than 2.0, with GFI = 0.915, AGFI = 0.891, NFI = 0.906, TLI = 0.908, and CFI = 0.922, which were affected less by the sample size and showed good suitability above 0.90, which was a good standard of fitness except that AGFI and RMSEA. In the study model, RMSEA = 0.060 showed a relatively direct effect. Seventeen paths out of the 24 suggested in the hypothetical model of this study were found to be significant; the 17 supported paths and final model are shown in Figure 3. The standardized estimation, CR and SMC revealed standardized direct, indirect, and total effect for the hypothetical model shown in Table 4.

**Figure 4:**
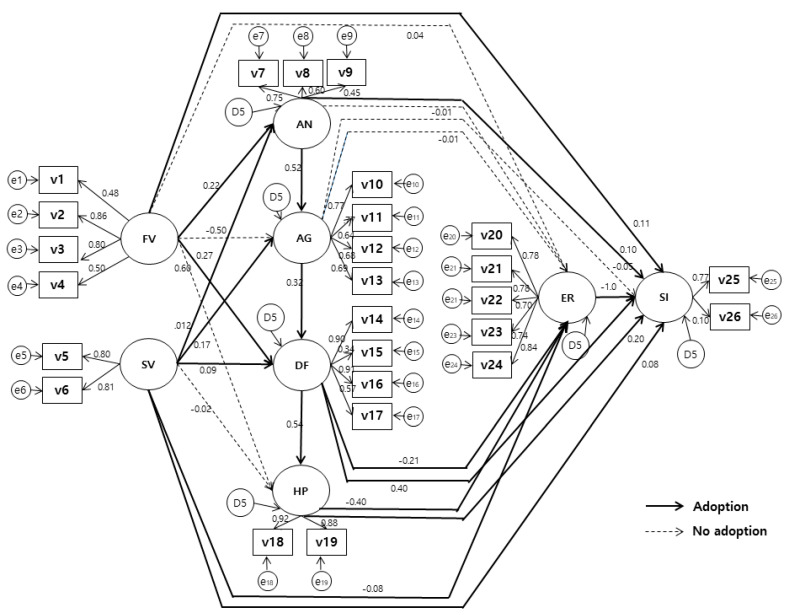
The path diagram of the hypothetical model. FV—Family violence-exposure; SV—School violence-exposure; AN—Anger; AG—Aggression; DF—Depression; HP—Hopelessness; ER—Ego-resilience; SI—Suicidal ideation; V1—Psychological violence witnessed; V2—Physical violence witnessed, V3—Verbal abuse; V4—Physical abuse; V5—School violence exposure 1; V6—School violence exposure 2; V7—Anger temperament; V8—Anger Reaction; V9—State anger; V10—Proactive-overt; V11—Reactive-overt; VX12—Proactive-relation; V13—Reactive-relational; V14—Emotional depression; V15—Positive emotion; V16—Isolation; V17—Physical deterioration; V18—Hopelessness 1; V19—Hopelessness 2; V20—Interpersonal; V21: Viability; V22—Emotional control; V23—Curiosity; V24—Optimism; V25—Suicidal ideation 1; V26—Suicidal ideation 2.

As a result of examining the skewness and kurtosis to confirm the normality of the distribution of each variable, the reference of substantial departure from normality as an absolute skew value closer to zero, we found that the kurtosis should be 3 for a perfect normal distribution. In findings, some of the skewness values of domestic violence, school violence, anger, and aggression exceeded 3, and the absolute value of the kurtosis value exceeded 10, but the rest of the variables were not exceeded. Accordingly, the data were analyzed after solving the problem by bootstrapping. (Figure 4).

Those that had a direct effect on suicidal ideation factors were depression at 0.40, feelings of hopelessness at 0.20, exposure to domestic violence at 0.11, self-resilience at −0.10, anger at 0.10, and exposure to school violence at 0.08, and all were statistically significant. Depression was 0.53 of the total effect, which was the largest factor in suicidal thoughts among all variables and was statistically significant. The terms of direct effect on self-elasticity, despair was the largest factor with −0.40, followed by depression at −0.21, and school violence exposure at −0.08, all of which were statistically significant.

The direct effect on anger was 0.22 in domestic violence exposure and 0.12 in school violence and3was statistically significant. The direct effect on aggression was 0.52 in anger and 0.17 in school violence, which were statistically significant. The direct effect on depression was 0.32 for aggression, 0.27 for domestic violence exposure, and 0.09 for school violence, all of which were statistically significant. As for the direct effect on feelings of hopelessness, depression was the largest factor with 0.54, and other variables were not statistically significant.

The indirect effect on suicidal thoughts was that domestic violence exposure was the largest factor with 0.18, followed by depression at 0.13, school violence exposure at 0.09, anger at 0.06 and aggression at 0.06, and all the findings were showed statistically significant correlation. The indirect effects on self-resilience were in the order of domestic violence exposure at −0.08, aggression at −0.06, school violence exposure at −0.03, anger at −0.03, and depression at −0.01, all of which had negative effects and were statistically significant. There was no variable with an indirect effect on anger, and the indirect effect on aggression was found to be 0.11 for domestic violence exposure and 0.06 for school violence, all of which were statistically significant. The indirect effect on depression was 0.07 for school violence exposure and 0.02 for domestic violence exposure, both of which were statistically significant. As for the indirect effect on feelings of hopelessness, domestic violence exposure was the largest factor at 0.16, and school violence exposure was statistically significant with 0.09.

## 4. Discussion

This study analyzed the direct effect of family and school violence exposure on adolescents’ suicidal ideation, leading to suicidal ideation through mediation of anger, aggression, depression, hopelessness, and ego resilience as negative factors.

First, the family violence exposure and suicidal ideation paths showed that most of the adolescents were exposed to family violence. Family violence exposure, such as child abuse by parents and the witness of parental violence of children, leads to suicidal ideation and risk of suicide risks among adolescents [33], However, there was evidence of physical, sexual, and emotional abuse from parents, such as pain, anger, and aggression by family members, leading to depression [34], which had a mediated effect on hopelessness to induce suicide [35]. It was predicted to be one of the variables to reduce suicidal ideation by mediating ego resilience [36]. In addition, this study also found that family violence exposure had significant effects on suicidal ideation such as anger and depression. Previous studies reported that characteristic anger predicts aggressive behavior in hostile situations [37]. However, there may be conflict and pain in doing so, and depressive tendency is sustained when physical abuse continues [30]. Hong [36] found that family violence exposure did not affect suicidal thoughts through mediation of hopelessness. In this study, exposure to family violence did not have a significant effect on suicidal ideation through the mediation of ego-resilience. A study reported that they play a role in buffering the relationship, but not in buffering suicidal ideation caused by interpersonal stress [24]. In a comparison of middle school and high school students, research reported that parental abuse is more frequent in middle school [18], and that middle school students have a greater tendency towards suicidal ideation. In addition, as high school students are significantly influenced by resilience and social support, it is suggested that more attention is paid to suicide risk of middle school students.

Second, because of school violence exposure and suicidal ideation pathways, most adolescents were exposed to school violence and thoughts of suicide. School violence exposure is one of the tension factors in school, and includes peer harassment as well as physical, verbal, and cyber bullying, showing a high direct relationship with youths’ suicidal ideation [38]. In addition, it was found that the experience of school violence damages mental health, leading to anger, aggression, and depression in adolescents. The higher levels of school violence led to high levels of anger and aggression [5]. However, this study was inconsistent with previous studies reporting that school violence, such as peer harassment, was highly associated with hopelessness. This was explained in Hong’s [36] study, which reported no mediating effects of hopelessness. Therefore, it is very important to inspect or provide psychological counseling whenever it is recognized and pointed out. In addition, this study found that school violence exposure directly affects anger, aggression, depression, and self-elasticity, and affects suicidal ideation through mediating ego resilience [38]. In view of the direct and indirect impacts, it is urgent to provide active practical intervention and supplementary measures rather than to prevent school violence [14,18].

Some of the previous studies reported that, suicidal thoughts and suicidal risk in adolescents occur through exposure to domestic violence, such as parental child abuse and child’s parental violence, which are tension factors at home [33,36]. However, in previous studies, physical, sexual, and emotional abuse from parents shows pain, anger, and aggression in the heart, and abuse from family leads to depression [35] and mediates feelings of despair to induce suicidal thoughts [39]. It has been found that the experience of school violence damages mental health in cases involving as anger, aggression, and depression of adolescents. The higher the level of one’s experience of damage from school violence, the higher the level of anger [24] and aggression expressed [26], which also has a significant effect on depression or leads to a high probability of developing depression [33,38]. The results were consistent with the findings of this study.

Third, in the pathway from family violence and school violence exposure to suicidal ideation, the results showed that family violence exposure directly affected anger and depression, but not aggression and hopelessness. Suicide is a self-destructive behavior in which anger is directed inward. High levels of anger have been identified as important predictors of suicide in young people, as well as depression [39]. However, aggression showed highly positive correlation with anger, but aggression was not accompanied by anger. Both family and school violence exposure did not directly affect hopelessness [22]. Hopelessness could not change the feelings of failure, depression, guilt, and misery, and both family and school violence exposure did not directly affect hopelessness. It is the most important psychological factor in predicting suicide ideation and behaviors [40]. In this study, hopelessness was not directly affected by violent exposure, but it was directly influenced by depression and had a significant effect on suicidal ideation. In addition, since violence exposure directly affects suicidal ideation, it is essential to eradicate family violence through parental involvement and self-resilience. Therefore, on the basis of the results of this study, it is necessary to develop intervention strategies regarding suicide ideation for middle school students that could be established and actively implemented to prevent adolescents’ suicide.

## 5. Conclusions

This study attempted to construct a structural model for predicting suicide risk through suicidal thoughts of middle school adolescents by identifying psychosocial and empirical factors that predict suicidal ideation of middle school students exposed to violence. Eight variables were generated through previous studies and literature reviews. As a result, family violence exposure was found to have a direct effect on anger, depression, self-elasticity, and suicidal ideation. School violence exposure has been found to have a direct effect on anger, aggression, depression, self-elasticity, and suicidal ideation. Anger, depression, and hopelessness had a direct effect on suicidal ideation, but ego resilience did not have a direct effect on suicidal ideation. This study suggests that, necessary to develop supportive intervention programs for family-oriented support for the adolescents and parents rather than strengthening their capacities.

## 6. Implications

First, in this study, the effect of self-resilience as a conditional factor to alleviate the suicidal thoughts of adolescents caused by tension factors such as domestic and school violence exposure was investigated. It is believed that this is necessary. Until now, most studies on suicide have been conducted on risk factors, and studies on protection factors have often only looked at direct effects. Therefore, follow-up studies are needed to come up with various in-depth strategies to prevent adolescent suicide from a multidimensional perspective.

Second, according to the results, anger and depression were significant mediators for suicidal thoughts in terms of domestic violence exposure, and anger, aggression, depression, and self-resilience were significant mediators in terms of school violence exposure. Based on the significant mediators identified in the research results, we propose a method for screening suicide risk groups and developing an intervention program. Rather than strengthening the ability, it is suggested that a supportive mediation program for active family members or support system interventions be developed.

## 7. Limitations

The study has some limitations. First, it focused only on middle school students among adolescents. In this study, since violence and suicide of adolescents were the main topics, we limited the focus to middle school students, a group predicted to have a high risk of violence and suicide during sample recruitment. Therefore, this research model is limited in application to out-of-school adolescents, high school students, and elementary school students corresponding to early youth. Therefore, it is suggested that the study is expanded to target various regions and include various related variables in order to build a model with higher predictive power in future research. 

## Figures and Tables

**Table 1:** Descriptive statistics of selected variables (*n* = 1150).

Variables	Categories	Min–Max	M ± SD	Skewness	Kurtosis	CR	AVE
Family violence exposure	Psychological violence witnessed	0–8	1.41 (1.96)	1.48	1.49	0.76	0.59
	Physical violence witnessed	0–11	0.67 (1.95)	3.54	12.61		
	Verbal abuse	0–15	1.59 (2.86)	2.41	5.99		
	physical abuse	0–10	2.86 (1.85)	2.27	4.97		
School violence * exposure	School violence exposure 1	0–4	0.22 (0.66)	3.67	14.22	0.81	0.71
	School violence exposure 2	0–3	0.31 (0.66)	2.17	4.05		
Anger	Anger temperament	5–20	9.94 (2.98)	0.74	0.71	0.79	0.63
	Anger reaction	5–20	9.49 (3.03)	0.78	0.53		
	State anger	10–40	11.83 (4.39)	3.51	13.96		
Aggression	Proactive–overt	10–20	14.05 (3.40)	1.61	3.71	0.85	0.73
	Reactive–overt	10–20	12.11 (4.43)	2.57	7.43		
	Proactive–relational	10–20	12.13 (2.98)	2.87	12.80		
	Reactive–relational	10–20	11.94 (3.14)	2.96	12.67		
Depression	Emotional depression	0–24	6.34 (5.00)	0.98	0.78	0.87	0.75
	Positive emotion	0–12	6.13 (2.82)	−0.12	−0.47		
	Isolation	0–18	4.17 (3.62)	1.07	0.97		
	Physical deterioration	0–6	1.46 (1.39)	0.94	0.55		
Hopelessness *	Hopelessness 1	0–10	2.32 (2.49)	1.14	0.55	0.89	0.81
	Hopelessness 2	0–10	2.46 (2.53)	1.04	0.27		
Ego-resilience	Interpersonal	3–15	10.47 (2.33)	−0.44	0.77	0.78	0.61
	Viability	2–10	7.00 (1.70)	−0.33	0.27		
	Emotional control	2–10	6.42 (1.72)	−0.01	0.07		
	Curiosity	5–25	17.10 (4.15)	−0.29	0.09		
	Optimism	2–10	6.43 (1.81)	−0.09	−0.04		
Suicidal ideation *	Suicidal ideation 1	8–22	10.49 (1.78)	1.20	0.98	0.88	0.76
	Suicidal ideation 2	9–25	12.84 (1.28)	1.20	1.45		

* School violence exposure, hopelessness, suicidal ideation used in item parceling method (divided in to 1 and 2). CR = construct reliability; AVE = average variance explained.

**Table 2:** Correlation of variables (*n* = 1150).

	V1	V2	V3	V4	V5	V6	V7	V8
V1	1							
V2	0.282 **	1						
V3	0.211 **	0.202 **	1					
V4	0.146 **	0.227 **	0.463 **	1				
V5	0.299 **	0.223 **	0.465 **	0.305 **	1			
V6	0.201 **	0.117 **	0.308 **	0.234 **	0.547 **	1		
V7	−0.080 *	−0.096 **	−0.135 **	−0.096 **	−0.273 **	−0.355 **	1	
V8	0.311 **	0.248 **	0.389 **	0.222 **	0.568 **	0.420 **	−0.225 **	1

* *p* < 0.05, ** *p* < 0.01. V1: family violence exposure, V2: school violence exposure, V3: anger, V4: aggression, V5: depression, V6: hopelessness, V7: ego resilience, V8: suicidal ideation.

**Table 3:** Fitness statistics of confirmatory factor analysis model (*n* = 1150).

Goodness	χ^2^ (*p*)	DF	CMIN/DF	GFI	AGFI	NFI	TLI	RMSEA	CFI	RMR
Criteria			≤5	≥0.9	≥0.9	≥0.9	≥0.9	≤0.08	≥0.9	≤0.08
Hypo Model	1325.245 (*p* < 0.01)	272	4.872	0.921	0.898	0.912	0.915	0.058	0.929	0.554

* RMSEA: Root Mean Squared Error Approximation, CMIN/DF: Minimum Discrepancy Per Degree Of Freedom, GFI: Goodness-Of-Fit Index (GFI), AGFI: Adjusted Goodness-Of-Fit Index, NFI: Normed Fit Index, NNFI: non-normed fit index and CFI: Comparative Fit Index.

**Table 4:** Standardized estimates, construct reliability (CR); Squared multiple correlation (SMC); and standardized direct, indirect, and total effect for the hypothetical model (*n* = 1150).

Endogenous Variable	Predictor Variable	S.E.	C.R. (*p*)	SMC	Direct Effect (*p*)	Indirect Effect (*p*)	Total Effect (*p*)	Adoption
Anger	Family violence	0.09	5.35 (0.002)	0.08	0.22 (0.002)		0.22 (0.002)	Yes
	School violence	0.17	2.84 (0.006)		0.12 (0.006)		0.12 (0.006)	Yes
Aggression	Family violence	0.09	1.56 (0.124)	0.31	−0.05 (0.124)	0.11 (0.002)	0.06 (0.084)	No
	School violence	0.17	4.87 (0.001)		0.17 (0.001)	0.06 (0.001)	0.23 (0.002)	Yes
	Anger	0.46	13.18 (0.003)		0.52 (0.003)		0.52 (0.002)	Yes
Depression	Family violence	0.17	7.45 (0.002)	0.24	0.27 (0.002)	0.02 (0.003)	0.29 (0.002)	Yes
	School violence	0.30	2.45 (0.032)		0.09 (0.030)	0.07 (0.002)	0.16 (0.002)	Yes
	Aggression	0.05	10.04 (0.002)		0.32 (0.002)		0.32 (0.002)	Yes
Hopelessness	Family violence	0.07	1.64 (0.133)	0.31	0.05 (0.133)	0.16 (0.003)	0.21 (0.003)	No
	School violence	0.14	0.77 (0.398)		−0.03 (0.398)	0.09 (0.002)	0.06 (0.002)	No
	Depression	0.02	15.96 (0.003)		0.54 (0.003)		0.54 (0.003)	Yes
Ego resilience	Family violence	0.07	−0.942 (0.279)	0.17	0.04 (0.279)	−0.08 (0.002)	−0.04 (0.260)	No
	School violence	0.13	−2.08 (0.038)		−0.08 (0.022)	−0.03 (0.002)	−0.11 (0.008)	Yes
	Anger	0.04	−0.05 (0.947)		−0.01 (0.947)	−0.03 (0.002)	−0.04 (0.002)	No
	Aggression	0.03	−0.14 (0.833)		−0.01 (0.833)	−0.06 (0.002)	−0.07 (0.002)	No
	Depression	0.02	−2.19 (0.028)		−0.21 (0.010)	−0.01 (0.002)	−0.22 (0.002)	Yes
	Hopelessness	0.03	−9.99 (0.002)		−0.40 (0.002)		−0.40 (0.002)	Yes
Suicidal ideation	Family violence	0.06	3.56 (0.005)	0.45	0.11 (0.005)	0.18 (0.002)	0.29 (0.002)	Yes
	School violence	0.11	2.67 (0.005)		0.08 (0.005)	0.09 (0.002)	0.17 (0.002)	Yes
	Anger	0.03	2.96 (0.004)		0.10 (0.004)	0.06 (0.002)	0.16 (0.002)	Yes
	Aggression	0.03	−1.58 (0.123)		−0.05 (0.123)	0.06 (0.001)	0.12 (0.002)	No
	Depression	0.01	11.49 (0.001)		0.40 (0.001)	0.13 (0.003)	0.53 (0.002)	Yes
	Hopelessness	0.02	6.23 (0.002)		0.20 (0.002)	0.04 (0.002)	0.24 (0.003)	Yes
	Ego resilience	0.03	−3.61 (0.002)		−0.10 (0.002)		−0.10 (0.002)	Yes

## Data Availability

Not applicable.
